# Global changes in chromatin accessibility and transcription in growth hormone-secreting pituitary adenoma

**DOI:** 10.1007/s12020-022-03155-z

**Published:** 2022-08-10

**Authors:** Meng Wang, Chenxing Ji, Yichao Zhang, Zhiqiang Zhang, Yu Zhang, Huiping Guo, Nidan Qiao, Xiang Zhou, Xiaoyun Cao, Zhen Ye, Yifei Yu, Vladimir Melnikov, Wei Gong, Min He, Zhaoyun Zhang, Yao Zhao, Xuelong Wang, Gang Wei, Zhao Ye

**Affiliations:** 1 Department of Endocrinology and Metabolism, Huashan Hospital, Shanghai Medical College, Fudan University, Shanghai, China; 2 Department of Neurosurgery, Huashan Hospital, Shanghai Medical College, Fudan University, National Center for Neurological Disorders, Shanghai, China; 3 CAS Key Laboratory of Computational Biology, Shanghai Institute of Nutrition and Health, University of Chinese Academy of Sciences, Chinese Academy of Sciences, Shanghai, China; 4 School of Life Sciences and Technology, Tongji University, Shanghai, China; 5 Shanghai Clinical Medical Center of Neurosurgery, Shanghai, China; 6 Shanghai Key Laboratory of Brain Function Restoration and Neural Regeneration, Shanghai, China; 7 National Clinical Research Center for Aging and Medicine, Huashan Hospital, Fudan University, Shanghai, China; 8 Sate Key Laboratory of Medical Neurobiology and MOE Frontiers Center for Brain Science, Institutes of Brain Science, Fudan University, Shanghai, China; 9 National Center for Neurological Disorders, Shanghai, China; 10 Neurosurgical Institute of Fudan University, Fudan University, Shanghai, China

**Keywords:** Growth hormone-secreting pituitary adenoma, Chromatin accessibility, RNA-seq, ATAC-seq

## Abstract

**Purpose:**

Growth hormone-secreting pituitary adenoma (GHPA) is an insidious disease with persistent hypersecretion of growth hormone and insulin-like growth factor 1, causing increased morbidity and mortality. Previous studies have investigated the transcription of GHPA. However, the gene regulatory landscape has not been fully characterized. The objective of our study was to unravel the changes in chromatin accessibility and transcription in GHPA.

**Methods:**

Six patients diagnosed with GHPA in the Department of Neurosurgery at Huashan Hospital were enrolled in our study. Primary pituitary adenoma tissues and adjacent normal pituitary specimens with no morphologic abnormalities from these six patients were obtained at surgery. RNA sequencing (RNA-seq) and assay for transposase-accessible chromatin with high-throughput sequencing (ATAC-seq) were applied to investigate the underlying relationship between gene expression and chromatin accessibility changes in GHPA.

**Results:**

Totally, 1528 differential expression genes (DEGs) were identified by transcriptomics analyses, including 725 up-regulated and 803 down-regulated. Further, we obtained 64 significantly DEGs including 10 DEGs were elevated and 54 DEGs were negligibly expressed in tumors tissues. The up-regulated DEGs were mainly involved in terms related to synapse formation, nervous system development and secretory pathway. In parallel, 3916 increased and 2895 decreased chromatin-accessible regions were mapped by ATAC-seq. Additionally, the chromatin accessible changes were frequently located adjacent to transcription factor CTCF and Rfx2 binding site.

**Conclusions:**

Our results are the first to demonstrate the landscape of chromatin accessibility in GHPA, which may contribute to illustrate the underlying transcriptional regulation mechanism of this disease.

## Introduction

Growth hormone-secreting pituitary adenoma (GHPA) is characterized by increased growth hormone (GH) and insulin-like growth factor I (IGF-1) secretion [1]. It can cause disproportionate skeletal, tissue, and organ growth, which are associated with significant morbidity and higher mortality [2]. Notably, in the last decade, although effective treatments have been largely improved for GHPA, including advanced surgical procedure, development of new radiotherapy techniques and availability of new medical therapies, ~40% proportion of patients still cannot completely achieve normal levels of GH and IGF-1 [3].

It is undeniable that alterations in mountainous genes have been involved in the onset and development of GHPA. Mutations in the α-subunit of the heterotrimeric Gs (*Gsα*), the stimulatory G protein (*GNAS*), α-subunit of protein kinase C (*PKC*) and aryl hydrocarbon receptor-interacting protein (*AIP*) have been discovered to be associated with GHPA [4–7]. Furthermore, multiplatform analysis has been performed to identify the epigenomic underpinnings of GHPA, which reveals that hypomethylation of promoter regions would increase the expression of *GH1*, somatostatin receptor 5 (*SSTR5*) and programmed death ligand 1 (*PD-L1*) in GHPA [8]. On the contrary, genes like high-mobility group A1 (*HMGA1*), *HMGA2*, and E2F Transcription Factor 1 (*E2F1*) have been reported to be down-regulated in GHPA [9]. The complex genetic network in GHPA allows for better understanding of tumor pathology. However, information on expression levels of somatotropinoma genes and how these are specifically regulated are still limited. Thus, exploring the regulatory elements of genes and their corresponding transcription factors (TFs) is critically important for elucidating the process of GHPA.

As a dynamic central regulator of transcription, chromatin is compacted in the nucleus and restricts the access of TFs to cis-regulatory elements [10]. In order to successfully interact with cis-regulatory elements, such as promoters and enhancers, TFs must induce chromatin remodeling of nucleosomal structures to result in different levels of chromatin accessibility [10]. Specifically, open chromatin regions allow transcription machinery components to access cis-regulatory elements and activate gene transcription, while closed chromatin regions impair the accessibility of promoters and enhancers to transcription factors and induce gene silencing [11]. Up to now, chromatin accessibility has been investigated in various diseases, which largely facilitates the acquisition of gene expression [12]. However, the role of chromatin remodeling in GHPA has not been investigated yet.

Assay for transposase-accessible chromatin with high-throughput sequencing (ATAC-seq) has been regarded as a robust and sensitive method for mapping genome-wide chromatin accessibility and TFs binding [13]. This is because during the analysis of ATAC-seq, DNA accessibility is probed with a hyperactive Tn5 transposase, which allows to simultaneously fragment and integrate the sequencing adapters preferentially in functional regions with accessible chromatin [13]. ATAC-seq has been widely adopted to investigate the pathophysiologic mechanisms of various tumors [14, 15].

In this study, we firstly utilized both ATAC-seq and mRNA-seq to investigate the pattern of genome-wide chromatin accessibility and characterize the chromatin accessibility landscape in GHPA.

## Material and methods

### Collection and storage of tissue specimens

Patients with GHPA were recruited from the Department of Neurosurgery at Huashan Hospital, affiliated with Shanghai Medical School, Fudan University. The diagnosis of GHPA was based on clinical manifestations, endocrine laboratory tests, and imaging data. Primary pituitary adenoma tissues and adjacent normal pituitary specimens with no morphologic abnormalities were obtained at surgery. It was histologically confirmed with blinding to sample identity by at least two senior pathologists after surgical resection. Previous studies have demonstrated that the use of normal pituitary tissue obtained at surgery is the preferred control in order to avoid inconsistencies in the treatment of tissue in autopsy specimens [16, 17]. In addition, the influence of heterogeneous factors such as age, gender and other diseases can also be avoided.

Totally, six GHPA tumor tissues and six corresponding para-adenoma normal tissues were obtained and immediately stored in liquid nitrogen. Owing to the small sample size, number of cells available for testing, especially for normal tissues, was limited. Thus, in ATAC-seq library preparation, we set the tumor as two aliquots, each one containing three samples, as well as for para-adenoma normal tissues. This study was approved by the Ethics and Research Committees of Huashan Hospital, Fudan University. Tissue samples and corresponding clinical materials were obtained with the written consent from all patients.

### RNA extraction, library preparation, and sequencing

The frozen tissues were ground separately using a liquid nitrogen-cooled mortar and pestle. The pulverized powder was then transferred into 1.5 ml RNase free falcon tubes containing 1 ml TRIzol reagent (Invitrogen, USA). The homogenates were further purified using RNeasy Mini Kit (QIAGEN, Germany) and run on Agilent Bioanalyzer 2100 to asses RNA integrity. The mRNAseq library preparation from 1 μg of total RNA was performed with TruSeq RNA Sample Prep Kit (Illumina, USA) according to the manufacturer’s instructions. 2 × 150 paired-end sequencing was performed on Illumina HiSeq X Ten platform (Novogene Biotech, China).

### Assay for transposase-accessible chromatin with high-throughput sequencing (ATAC-seq)

The ATAC-seq libraries were prepared as previously described [18, 19]. Briefly, the frozen tissues were ground separately using a liquid nitrogen-cooled mortar and pestle. The pulverized powder was then transferred into 1.5 ml falcon tubes containing lysis buffer (10 mM Tris-HCl at pH7.4, 10 mM NaCl, 3 mM MgCl_2_ and 0.1% IGEPAL CA-630 [Sigma-Aldrich]) for 10 min on ice to prepare the nuclei. Immediately after lysis, nuclei were spun at 500 × *g* for 5 min to remove the supernatant. Nuclei were then incubated with the Tn5 transposome (Vazyme Biotech, China) and tagmentation buffer at 37 °C for 30 min. After the tagmentation, the stop buffer was added directly into the reaction to end the tagmentation. PCR was performed to amplify the library for 10–12 cycles using the following PCR conditions: 72 °C for 3 min; 98 °C for 30 s; and thermocycling at 98 °C for 15 s, 60 °C for 30 s and 72 °C for 3 min; following by 72 °C 5 min. After the PCR reaction, libraries were purified with Agencourt Ampure XP beads (Beckman, USA) to remove contaminating primer dimers and quantitated using Qubit 2.0 Fluorimeter (Invitrogen, USA). All libraries were sequenced on the Illumina HiSeq X Ten with 150 bp paired-end reads (Novogene Biotech, China).

### mRNA-seq data processing

The quality control of mRNA sequencing data was performed via FastQC (http://www.bioinformatics.babraham.ac.uk/projects/fastqc/). Reads from RNA-seq experiments were aligned to human genome build hg19 using STAR [20]. The mRNA expression of a gene was quantified by fragments per kilobase million reads (FPKM) based on RefSeq gene annotation using Cuffdiff [21]. Differential expression genes (DEGs) were identified by a *p* value < 0.05, fold change (FC) > 1.5 and the average FPKM > 5 for at least one of the two groups. The aligned BAM files were converted to bigWig format and normalized by reads per kilobase million (RPKM) with the bamCoverage script in deepTools 2.0 [22]. The deepTools 2.0 and ggplot2 [23] from R package were used for data normalization and visualization.

### ATAC-seq data processing

The quality of sequenced ATAC-seq data was evaluated with FastQC (http://www.bioinformatics.babraham.ac.uk/projects/fastqc/). All sequencing data were mapped onto the hg19 human genome assembly using Bowtie2 [24] with an insert size of less than or equal to 2000 bp (command line parameter -X 2000). Duplicate reads were removed with SAMtools [25], only non-duplicate reads kept in the BAM format were used for the subsequent analysis. MACS2 [26] was used to identify regions of ATAC-seq peaks (with the parameters --shift -100 --extsize 200). Picard Tools (v.2.2.4, https://broadinstitute.github.io/picard/) and ggplot2 [23] were used to plot the distribution of paired-end sequencing fragment sizes. For visualization, the BAM files were converted to the bigWig format using the bamCoverage [22]. The heatmaps and average profiles were also generated using the plotHeatmap and plotProfile functions in deepTools.

### GO and KEGG pathway enrichment analysis

The Gene Ontology (GO) enrichment and Kyoto Encyclopedia of Genes and Genomes (KEGG) pathway analysis were performed using clusterProfiler [27], which is an R package for functional classification and enrichment of gene clusters using hypergeometric distribution. The enriched terms with *p* value< 0.05 were considered significant.

### Annotation of genomic regions

To annotate the location of ATAC-seq signal in terms of important genomic features, the annotatePeaks.pl script in HOMER [28] was used to assign the peak files in BED format to promoter-TSS (by default defined from −1 Kb to +100 bp of transcription start site), TTS (by default defined from −100 bp to +1 Kb of transcription termination site), Intron, Intergenic, Exon, etc. with default parameters.

### Differential analysis of chromatin accessibility and mRNA expression

For purposes of assessing the alteration of chromatin accessibility genome-wide, deepTools was used to compute the average scores (RPKM normalized values) for transposase hypersensitive sites in each sample. Hyper-accessible regions (hyper) associated with increased ATAC-seq signal were defined if the regions showed fold change (FC) > 1.5 in the tumor group as compared to the normal group. On the contrary, the hypo-accessible regions associated with decreased ATAC-seq signal were defined if the regions showed fold change >1.5 in normal group compared to tumor group. The heatmaps and average profiles of regions expanded to ±1.5 Kb surrounding differentially accessible regions (DARs) center were generated using the plotHeatmap and plotProfile functions in deepTools [22]. The annotation of DARs to genomic features was performed using the HOMER suite tool annotatePeaks [28], then the DARs neighboring genes were overlapped with DEGs from mRNA-seq data, so as to address the global relationship between the ATAC-seq signal at differentially accessible regions and the transcription levels of the corresponding DEGs.

### Transcription factor motif enrichment and occurrences analysis

The findMotifsGenome function in HOMER v4.9 [28] were used to analyze transcription factor (TF) motif enrichment of ATAC-seq peak summits (with option -size -200,200), which was also used to predict binding sites and target genes of top 50 enriched TFs. The annotatePeaks.plscript was used to identify the nearest genes of predicted TF binding sites.

### Statistical analysis and data visualization

If not specified, R platform (https://www.R-project.org/) was used to compute statistics and generate plots. Fisher’s exact test, Student’s *t* test, Pearson’s correlation coefficient, Wilcoxon signed rank test were used to assess the significance. Fold change, *p* values and false discovery rate were calculated in analysis. Significant differences for all quantitative data were considered when ^*^p < 0.05, ^**^p < 0.01, ^***^p < 0.001, and ^****^p < 0.0001. Genome browser images were made using the Integrative Genomics Viewer (IGV) [29] with bigWig files processed as described above.

## Results

### The characteristics of mRNA expression in GHPA

**Fig. 1:**
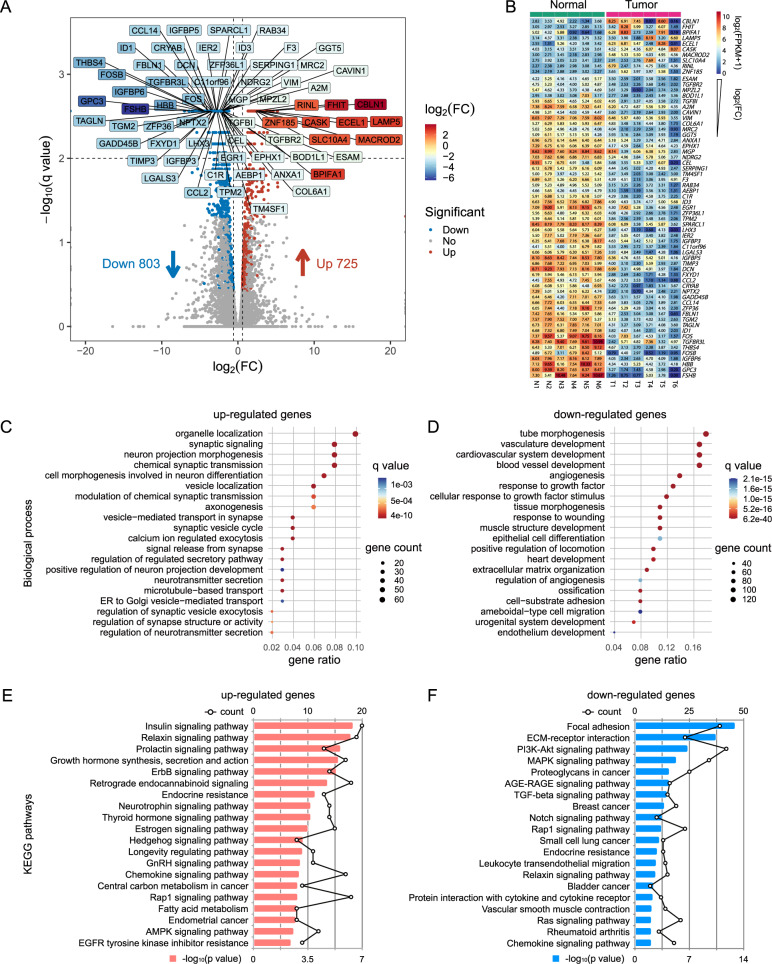
The characteristics of mRNA expression in GHPA. **A** Volcano plot shows the transcript levels differentially expressed genes (DEGs) between the para-adenoma normal pituitary tissues and pituitary tumors. 725 significantly up-regulated genes are shown as red dots, whereas 803 down-regulated genes are shown as blue dots. Filtered by *q* value < 0.05, |log_2_(FC) | ≥ 2 and average FPKM ≥ 10, 64 significantly differentially expressed key genes are labeled and shown in different colors according to log_2_(FC). **B** Heatmap of 64 key genes labeled in volcano plot (A), listed in a descending order based on log_2_(FC). The numeric values are log_2_(FPKM + 1). **C** Bubble plot showing GO enrichment analysis of upregulated genes. Top 20 GO terms of biological processes (BP) significantly enriched by up-regulated genes are depicted. **D** Bubble plot showing GO enrichment analysis of down-regulated genes. Top 20 GO terms of biological processes (BP) significantly enriched by down-regulated genes are depicted. **E** Bar plot showing top 20 KEGG pathways significantly enriched by upregulated genes. The polygonal chain in black shows the gene count related to each GO term. **F** Bar plot showing top 20 KEGG pathways significantly enriched by downregulated genes. The polygonal chain in black shows the gene count related to each GO term

To investigate gene expression profiles of GHPA, mRNA-seq was used to examine genotypic changes in tumors and adjacent normal pituitary tissues (Fig. S1A, B). A total of 1528 differential expression genes (DEGs) were identified using Cuffdiff [21], among which 725 genes were up-regulated and 803 genes were down-regulated (Fig. 1A, Fig. S1C, D). In order to reveal the crucial candidate genes associated with GHPA, we obtained 64 significantly DEGs, which were filtered by *q* value < 0.05, |log_2_fold change| ≥2 and average FPKM ≥ 10. As shown in Fig. 1B, 10 of these 64 DEGs, including *CBLN1*, *FHIT* and *BPIFA1*, were elevated in the tumors, and the other 54 DEGs were negligibly expressed, such as *ESAM*, *TGFBR2*, *ID1* and *FSHB*. Furthermore, GO enrichment analysis was performed to reveal the probable biological process, cellular components, and specific molecular functions of all DGEs. As shown in Fig. 1C, the up-regulated DEGs were mainly involved in terms related to synapse formation, nervous system development and secretory pathway. Meanwhile, the down-regulated DEGs were mainly relevant to cardiovascular system development, growth factor response and extracellular matrix organization (Fig. 1D). KEGG pathway enrichment analysis was consistent with the enrichment in GO terms. As shown in Fig. 1E, up-regulated DEGs were significantly enriched in various hormone-related signaling pathways (such as insulin, relaxin, prolactin, growth hormone, thyroid hormone, estrogen and GnRH signaling pathways) and proliferation-related signaling pathways (ErbB, neurotrophin, hedgehog, and chemokine signaling pathway). The downregulated DEGs largely enriched in focal adhesion, PI3K-Akt, MAPK, and TGF-beta signaling pathways (Fig. 1F).

### The accessible chromatin landscape of GHPA

**Fig. 2:**
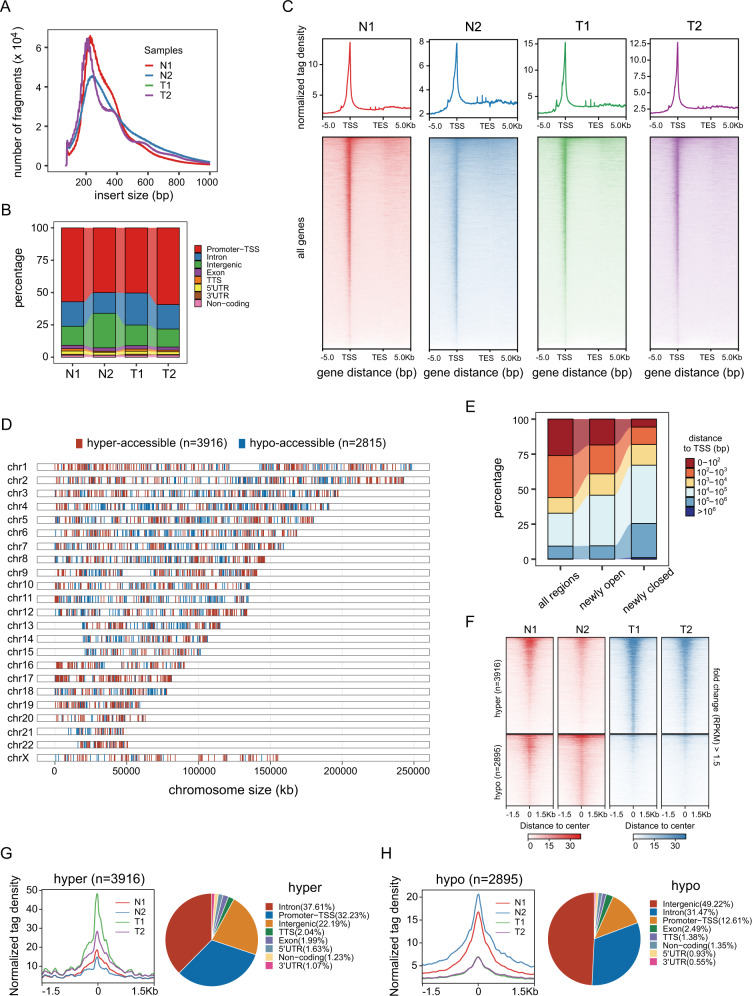
The accessible chromatin landscape of GHPA. **A** Distribution of ATAC-seq fragment size in each sample. **B** Annotation of ATAC-seq peaks to genomic features: exon, intergenic regions, introns, 3′ UTR, 5′ UTR, promoters-TSS, TES and no-coding regions. Peak summit located up to 1 Kb upstream and 100 bp downstream of the TSS are determined as promoter-TSS regions. **C** Average read density (top panel) and heatmaps (bottom panel) indicating ATAC-seq signal across a genomic window of 5 Kb upstream of the TSS and 5 Kb downstream of the TES. **D** Distribution of hyper- (red) and hypo-accessible (blue) regions over chromosomes in pituitary tumors compared with normal pituitary tissues. **E** Distance to closest transcription start site (TSS) of all accessible regions and differentially open and closed regions. **F** Heatmap representation of normalized ATAC-seq signal in the para-adenoma normal tissues and tumors tissues over DARs. The top set of panels show read signal over the 3916 hyper-accessible regions in pituitary tumors, while the bottom set of panels show read signal over the 2895 hypo-accessible regions. Signals within 1.5 Kb surrounding the center of DARs are displayed in a descending order. **G** Profiles of normalized tag density across a genomic window of ±1.5 Kb surrounding the center of hyper-accessible regions. Pie chart showing the proportion of these sites within the indicated genomic regions. **H** Profiles of normalized tag density across a genomic window of ±1.5 Kb surrounding the center of hypo-accessible regions. Pie chart showing the proportion of these sites within the indicated genomic regions

To gain insight into the transcriptional mechanisms of GHPA, ATAC­seq was performed to assess the chromatin accessibility landscape. As the chromatin could be fragmented into mono-, di- and tri-nucleosome patterns, the fragments distribution was similar in each of our sample (Fig. 2A). This indicated that chromatin was accessible to transposase in the same degree for all these samples. Furthermore, the genome-wide analysis of transposase hypersensitive sites distribution over functional genomic elements suggested that promoter-TSS, introns and intergenic regions were preferentially accessible to Tn5 transposase (Fig. 2B). Moreover, our result demonstrated that ATAC-seq signal was significantly enriched at ±1 Kb region surrounding the TSS (Fig. 2C). This conformed to the pattern of previous studies in multiple organisms and suggested that the majority of cis-regulatory regions were located in the vicinity of gene core promoters and that open chromatin at these sites was predictive of active transcription [30, 31]. In total, there were 6811 peaks of different accessible regions (DARs) been obtained (|fold change| > 1.5), including 3916 hyper-accessible open peaks and 2895 hypo-accessible close peaks (Fig. 2D). To better understand the potent role of the above DARs in regulating transcription, we calculated and classified the distances of these specific regions to TSS. Newly opened regions indicated a significant enrichment in 10^4^–10^5 ^bp away from TSS, while a shrink of hyper-accessible regions was displayed within 0–10^3 ^bp. On the contrary, regions with a distance beyond 10^4 ^bp to TSS, which were defined as newly closed, comprised an enormous part of all regions. An increased proportion of hypo-accessible regions was discovered with more than 1000 bp distance from TSS, which indirectly suggested distal cis-regulatory element like enhancer might be deactivated (Fig. 2E, F). Further analysis revealed that a majority of DARs distributed at the intron and distal intergenic regions, as shown in Fig. 2G, H, suggesting that DARs might represent distal regulatory elements such as enhancers.

### Association between gene expression and chromatin accessibility in GHPA

**Fig. 3:**
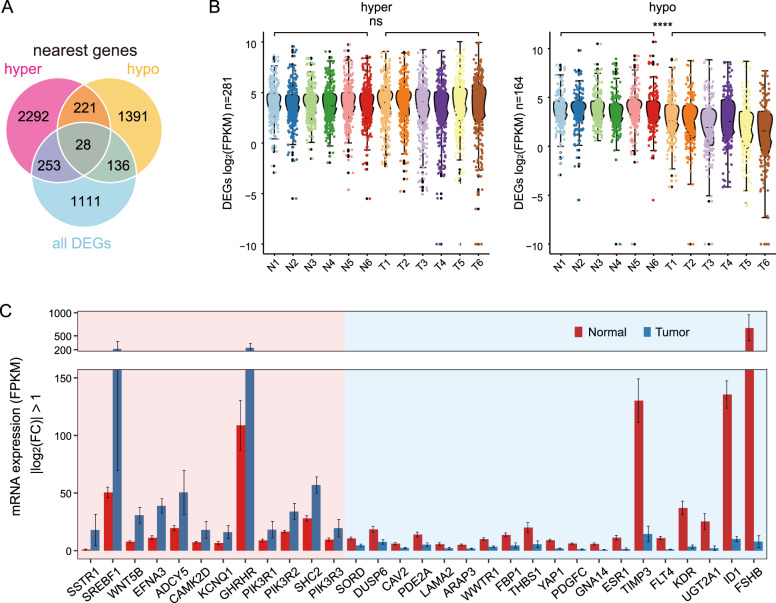
Filtration of key DEGs related to differentially accessible regions. **A** Venn diagram illustrating the overlap among all DEGs and nearest genes associated with the differentially accessible regions. **B** Box plots for mRNA expression levels of DEGs relative to hyper- (left panel) and (right panel) hypo-accessible regions. Notches of the boxes indicate medians. The *p* values are calculated by Wilcoxon’s signed-rank test. ^*^p < 0.05; ^**^p < 0.01^; ***^p < 0.001; ^****^p < 0.0001; ns, no significance. **C** Bar plot showing average expression levels (FPKM) of 31 key DEGs related to hyper- (red shade area) and hypo-accessible (blue shade area) regions. DEGs are listed in a descending order based on log_2_(FC)

To establish whether the alterations in gene expression changes associated with chromatin accessibility, we performed an integrated analysis with mRNA-seq and ATAC-seq data. By assigning differential accessible sites to their nearest genes, 281 and 164 DEGs were identified to be related to the hyper- and hypo-accessible regions respectively (Fig. 3A, Supplementary Tables 1, 2). Interestingly, most of the DEGs near hypo-accessible regions were significantly down-regulated (*p* < 0.0001), but the relationship between the hyper-accessible regions and their nearest DEGs was slight (Fig. 3B). It suggested that decreased chromatin accessibility contributed greatly to the down-regulation of genes associated with GHPA. Terminally, 31 key DEGs were identified to correlate with changes in chromatin accessibility, with |log_2_fold change| > 1. Genes like Somatostatin Receptor 1 (*SSTR1*), Sterol Regulatory Element Binding Transcription Factor 1 (*SREBF1*), Wnt Family Member 5B (*WNT5B*) and Growth Hormone Releasing Hormone Receptor (*GHRHR*) were significantly upregulated nearby the hyper-accessible regions. On the contrary, restrained chromatin accessibility diminished expression of several genes such as Sorbitol Dehydrogenase (*SORD*), Dual specificity phosphatase 6 (*DUSP6*), Laminin subunit alpha-2 (*LAMA2*), WW Domain Containing Transcription Regulator 1 (*WWTR1*, commonly listed as *TAZ*), Metalloproteinase inhibitor 3 (*TIMP3*), Yes1 Associated Transcriptional Regulator (*YAP1)* and Follicle Stimulating Hormone Subunit Beta (*FSHB*) (Fig. 3C).

**Fig. 4:**
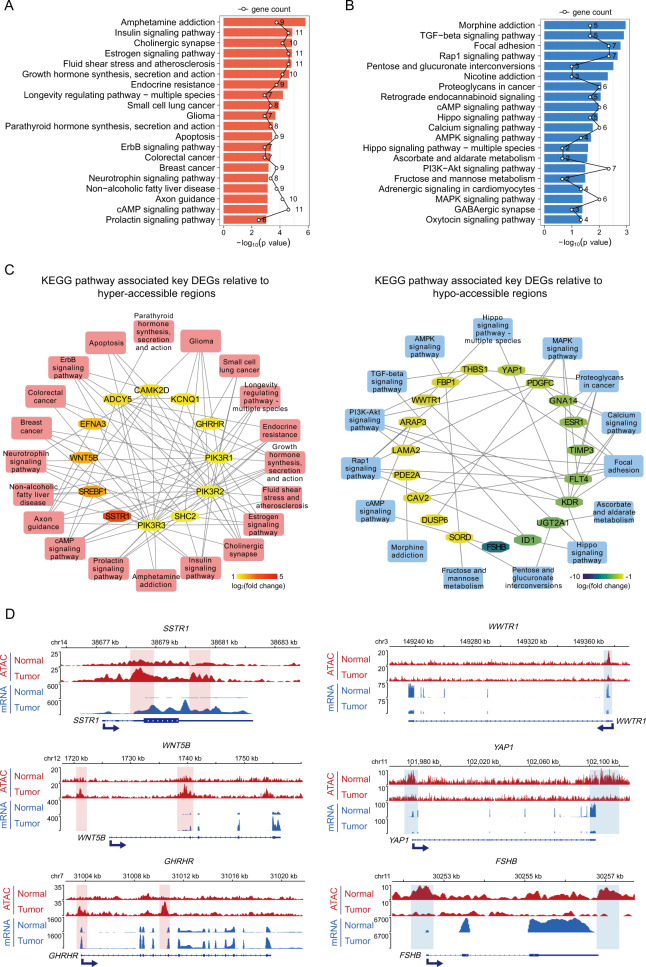
KEGG pathway associated key DEGs related to differentially accessible regions. **A** Top 20 KEGG pathway-associated DEGs related to hyper-accessible regions. The polygonal chain in black shows the gene count enriched in each KEGG pathway. **B** Top 20 KEGG pathway-associated DEGs related to hypo-accessible regions. The polygonal chain in black shows the gene count enriched in each KEGG pathway. **C** KEGG pathway associated key DEGs related to hyper- (left panel) and hypo-accessible (right panel) regions. Inner hexagons represent key DEGs and outside rectangles represent relative KEGG pathways. **D** Genomic snapshots of ATAC-seq (red) and mRNA-seq (blue) signal of representative (left panel) up-regulated and (right panel) down-regulated genes associated with key signal pathways and positively correlated to hyper- (red shade area) and hypo-accessible (blue shade area) regions, respectively

To deeply insight into the functional role and the latent signaling pathway of the DARs and DEGs, KEGG pathways analysis were performed. The DEGs associated with hyper-accessible regions were enriched in pathways relative to Insulin, Estrogen, GH, ErbB, Axon guidance, and cAMP signaling pathway (Fig. 4A). The activation of these pathways was implicated in proliferation, invasion, and GH secreting level of GHPA. Meanwhile, the DEGs associated with hypo-accessible regions might desensitize TGF-beta, Hippo and MAPK signaling pathway, which may trigger the proliferation and secretion of pituitary tumors in an inactive form (Fig. 4B). Next, as shown in Fig. 4C, we set out to identify the core genes in KEGG pathway networks relative to hyper- or hypo-accessible regions, respectively.

The increased chromatin accessibility at promoter-TSS regions or distal regulatory elements may contribute to significant up-regulation of *SSTR1*, *WNT5B,* and *GHRHR* (Fig. 4D, Fig. S2). Visualization of ATAC-seq signals and mRNA-seq expression levels at *GHRHR* locus revealed a significant accessibility peak enriched in *GHRHR* intron region and elevated mRNA expression compared with normal tissue. Hyper-accessible 5′-UTR at *SSTR1* in tumor contribute to the expression of *SSTR1* mRNA. The intergenic and intron regions at *WNT5B* have marked as two discrete peaks, which was accompanied with an enhanced expression of *WNT5B* in tumor. Meanwhile, down-regulation of genes, such as *WWTR1*, *YAP1,* and *FSHB*, were closely linked to nearby decreased chromatin accessibility (Fig. 4D, Fig. S2). Intervening in the accessibility of specific intron, intergenic or promoter-TSS regions might enervate chromatin accessibility, leading to attenuate these gene expressions.

### Prediction of transcription factors involved in chromatin accessibility changes between the para-adenoma normal tissues and GHPA

**Fig. 5:**
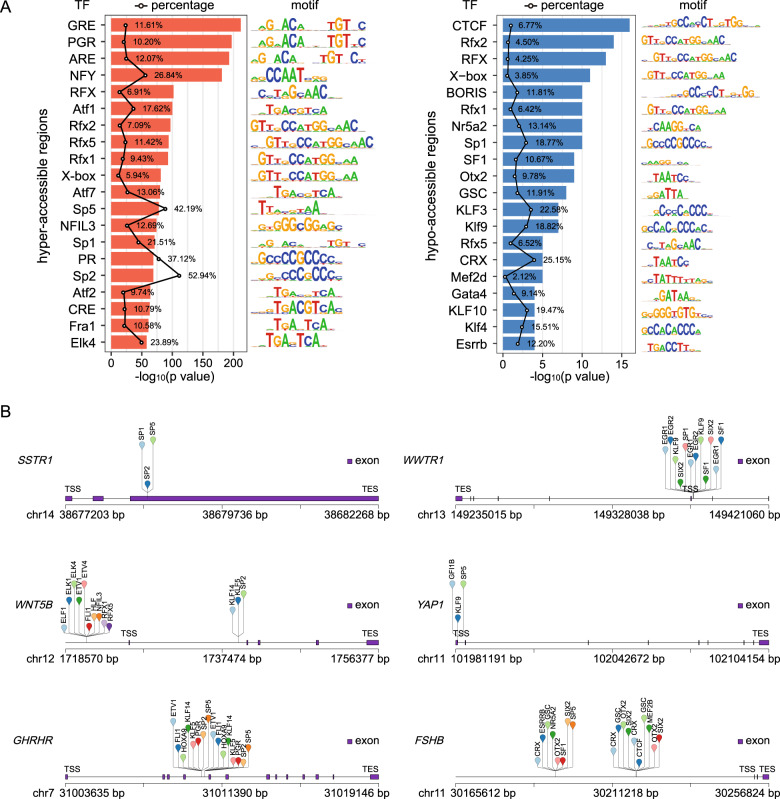
Prediction of transcription factors involved in chromatin accessibility changes between the para-adenoma normal tissues and GHPA. **A** Top 20 enriched known TF motifs of hyper- (left panel) and hypo-accessible (right panel) sites, with *p* values estimated from HOMER v4.9. The polygonal chain in black shows the percentages of target sequences with TF motif. **B** Putative TF occupancy of representative up- (left panel) and down-regulated (right panel) genes positively correlated to chromatin accessibility changes

Transcription factors (TFs) are proteins that regulate gene expression through binding to specific DNA sites in cis-regulatory regions of target genes [32]. Normally, chromatin accessibility to DNA is a prerequisite for the binding of TFs. Recently, numerous studies have revealed that TFs play an important role in promoting tumor metastasis and migration through ATAC-seq [33]. To examine potential TFs located in the differentially accessible regions between tumoral tissues and para-adenoma normal tissues, we searched for responsible TFs using motif discovery software HOMER [28]. As accessible chromatin sites are often obligated if TFs bind to their cognate DNA sequence motifs, the integration of TF motifs and chromatin accessibility data from ATAC-seq can predict TF occupancy on chromatin [18, 34]. Using the ±200 bp flanking sequences around summits of ATAC-seq peaks to identify motif occurrences and thus predict binding sites for TFs, we identified that 264 and 39 potential TFs were in hyper- and hypo-accessible regions respectively (*p* < 0.01, Supplementary Tables 3, 4). As shown in Fig. 5A, the hyper-accessible regions were highly enriched for GRE, PGR, ARE and evolutionarily conserved RFX family transcription factors, while the hypo-accessible regions were enriched for CTCF, RFX, and KLF family transcription factors. To obtain potential regulators of DEGs associated with GHPA, we performed the analysis of the binding sites of top 50 TFs enriched in DARs using HOMER (v4.10) [28] (Fig. 5B). The results showed that ETV1 and KLF14 potentially bound to the hyper-accessible sites of *GHRHR* and *WNT5B* gene body in GHPA, and the down-regulation of *FSHB, WWTR1* and *YAP1* might be closely correlated with the absence of CTCF, which functions as an insulator in chromatin reorganization and could mediate long-range enhancer-promoter interaction to regulate gene expression [35]. Altogether, our data demonstrated that the decreased chromatin accessibility, which led to the reduced binding capacity of critical TFs, might be responsible for the down-regulation of key DEGs associated with GHPA.

## Discussion

The genome-wide landscape of GHPA has been investigated in a few studies, but the variation of chromatin state in GHPA has never been investigated [8, 36]. As chromatin structure change exerts significant influence on gene transcription, it has become the “hot spot” in the study of pathological mechanism of various diseases [37, 38]. The commonly genome-wide chromatin accessibility profiling methodologies include MNase-seq, DNase-seq, FAIRE-seq and ATAC-seq, which can further analyze the alterations in the accessible chromatin state and understand the molecular mechanisms of several diseases [11, 39]. Due to its speed, simplicity, and low input cell number requirement, ATAC-seq has been implicated as an accurate and sensitive measure of genome-wide chromatin accessibility [40]. Moreover, ATAC-seq has also been widely adopted to predict TF binding motifs in these regulatory regions [41].

Here, based on the RNA-seq, 64 significantly DEGs were obtained, including 10 upregulated DEGs and 54 down-regulated DEGs, in GHPA compared with normal pituitary tissues. It is worth noting that most up-regulated DEGs in tumors participate in various hormone-related signaling pathways (such as insulin, prolactin, and growth hormone) and pathways in GH synthesis, secretion and action. GH secretion by the somatotroph cells is mediated by ghrelin and GH-releasing hormone (GHRH) and inhibited by somatostatin and IGF-1 feedback inhibition [42]. The effects of GH-releasing hormone are mediated by GHRH-receptor (GHRHR), a seven transmembrane G-protein coupled receptor which is expressed primarily on the cell surface of somatotrophs in the anterior pituitary [43]. Under physical conditions, GHRH specifically binding to GHRHR facilitates somatotroph cell growth, growth hormone gene transcription and growth hormone secretion [44]. Consistent with the previous result [45], our data show that the expression of *GHRHR* is significantly up-regulated in GHPA compared with normal pituitary tissues. Somatostatin binds to SSTR1-5 to inhibit GH secretion [46]. Previous studies have reported that SSTRs expression is increased in tumor tissue of GHPA patients [47]. Elevated expression of SSTRs has been considered as a valuable indicator in predicting biochemical response to somatostatin analogs treatment [48]. Here, our data show the expression of SSTR1 is up-regulated in GHPA compared with normal pituitary tissue. There are few previous studies in investigating the level of SSTR1 in GHPA. However, our results suggest that additional research is needed to uncover this issue, which may provide potential target for GHPA treatment.

Additionally, our data reveal that the down-regulated DGEs are enriched in signaling pathways that included focal adhesion pathway. Focal adhesion, a subcellular structure between the cell and the extracellular matrix, plays an important role in cell migration through tissues [49]. Previous studies have demonstrated that the invasion and metastasis of several malignant tumors, such as breast cancer and gastric cancer, are closely related to focal adhesion [50, 51]. From RNA-seq data, we find genes which encode collagen family are significantly down-regulated. Collagen is one of the essential components which participate in the formation of focal adhesion [52]. Suppression of collagen might breach focal adhesion and dramatically enhance the invasion of tumor cells [53, 54]. Therefore, we speculate that decreased collagen in GHPA may interfere with the formation of focal adhesion, leading to potential invasion of tumor.

Further, ATAC-seq were undertook to reveal the noticeable alterations in transcriptomes and chromatin accessibility of GHPA. Comparing with normal pituitary tissues, there are 64 significantly DEGs identified in GHPA, the proportion of down-regulated DEGs is higher than that of up-regulated DEGs, which is corresponding to previous studies [55, 56]. Combined with our ATAC-seq date, most of the DEGs near hypo-accessible regions are significantly down-regulated, which clearly suggests that decreased chromatin accessibility contributes to the down-regulation of genes. Furthermore, these hypo-accessible chromatin regions were rich in the motifs of critical regulatory factors, such as CTCF, Rfx2, and SF1. According to these data, we speculate that, by affecting the DNA-binding ability of TFs and transcriptional regulators in promoter and/or enhancer, the global decreases in chromatin accessibility could inhibit the expression of critical genes associated with GHPA. For example, it is probably that the significant decrease in chromatin openness leads to loss of chromatin insulator CTCF binding, which impairs the long-range interactions between promoters and enhancers of *FSHB*.

In conclusion, our study is the first to depicture global changes in chromatin accessibility and transcription in GHPA. Combination of these data can provide relevant insight into the tumor biology, especially in regulatory network that contributes to tumor formation and growth. Our results demonstrated that the decreased chromatin-accessible regions are depleted for TF binding consensus motifs, which will lead the expression decrease in GHPA terminally. However, we are aware of the limitations of this research. Firstly, the sample size of GHPA and control group is small. Secondly, more informative is needed to validate the present findings in vitro and in vivo. The mechanism and functional consequence behind the interactions between TFs and chromatin accessibility change regions is worth further investigation.

## Supplementary Information

Supplementary Figure legends

Figure S1

Figure S2

Supplementary Tables
