# Supplementary material for: Global changes in chromatin accessibility and transcription in growth hormone-secreting pituitary adenoma
Source: Endocrine. 2022 Aug 10;78(2):329–42. doi: 10.1007/s12020-022-03155-z (PMC9584994; doi:10.1007/s12020-022-03155-z)
Supplement: Supplementary file 1 — Supplementary Figure legends [file 12020_2022_3155_MOESM1_ESM.pdf]

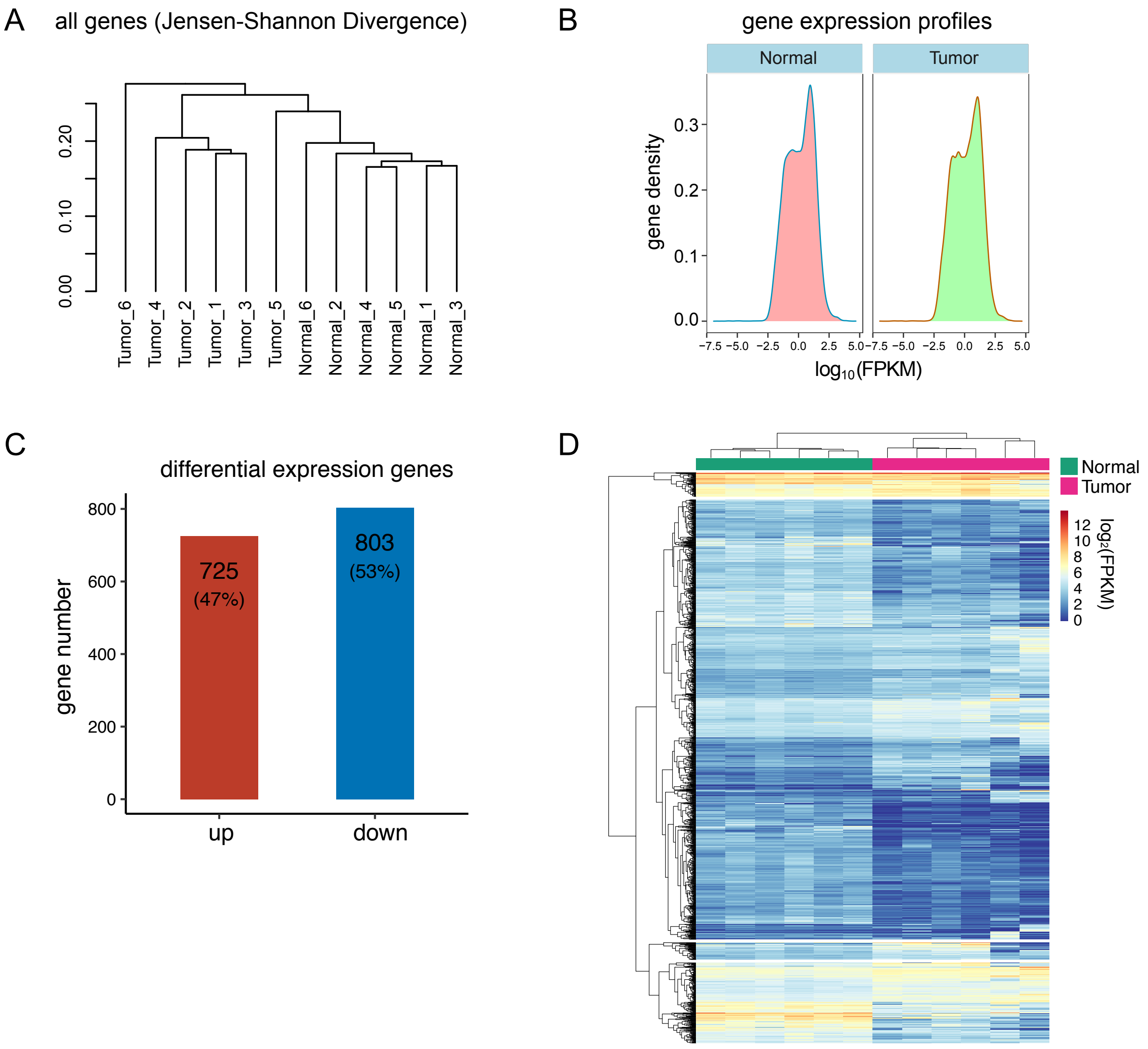
A
all genes (Jensen-Shannon Divergence)
0.00
0.10
0.20
Tumor_6
Tumor_4
Tumor_2
Tumor_1
Tumor_3
Tumor_5
Normal_6
Normal_2
Normal_4
Normal_5
Normal_1
Normal_3
B
gene expression profiles
Normal
Tumor
gene density
0.0
0.1
0.2
0.3
−7.5 −5.0 −2.5 0.0 2.5 5.0
−7.5 −5.0 −2.5 0.0 2.5 5.0
log10(FPKM)
C
differential expression genes
gene number
0
200
400
600
800
725
(47%)
803
(53%)
up
down
D
Normal
Tumor
12
10
8
6
4
2
0
log2(FPKM)
