# Supplementary figures and images for: Global changes in chromatin accessibility and transcription in growth hormone-secreting pituitary adenoma
Source: Endocrine. 2022 Aug 10;78(2):329–42. doi: 10.1007/s12020-022-03155-z (PMC9584994; doi:10.1007/s12020-022-03155-z)

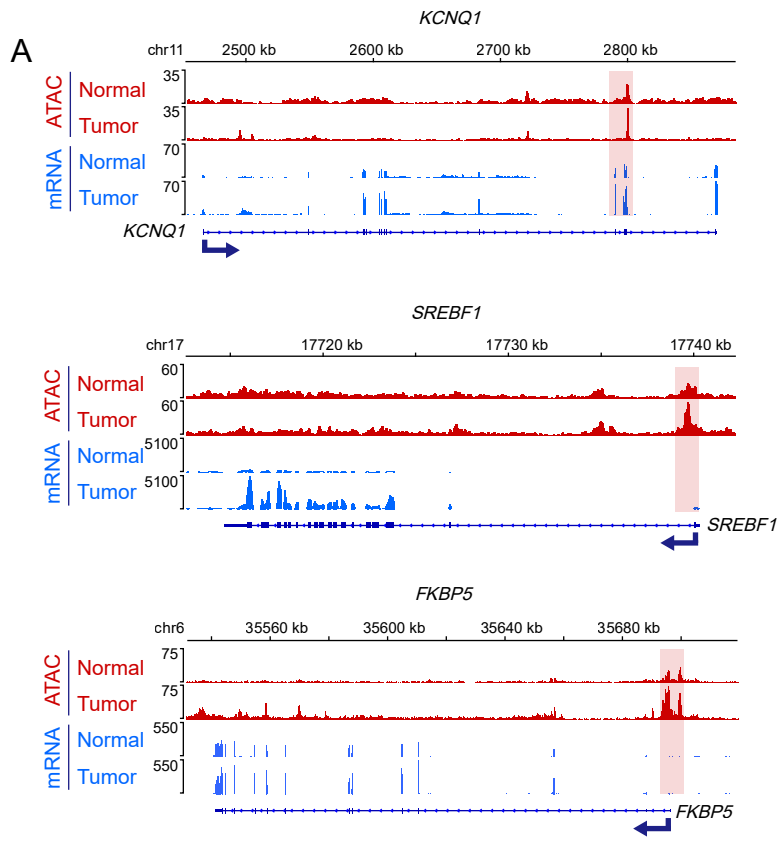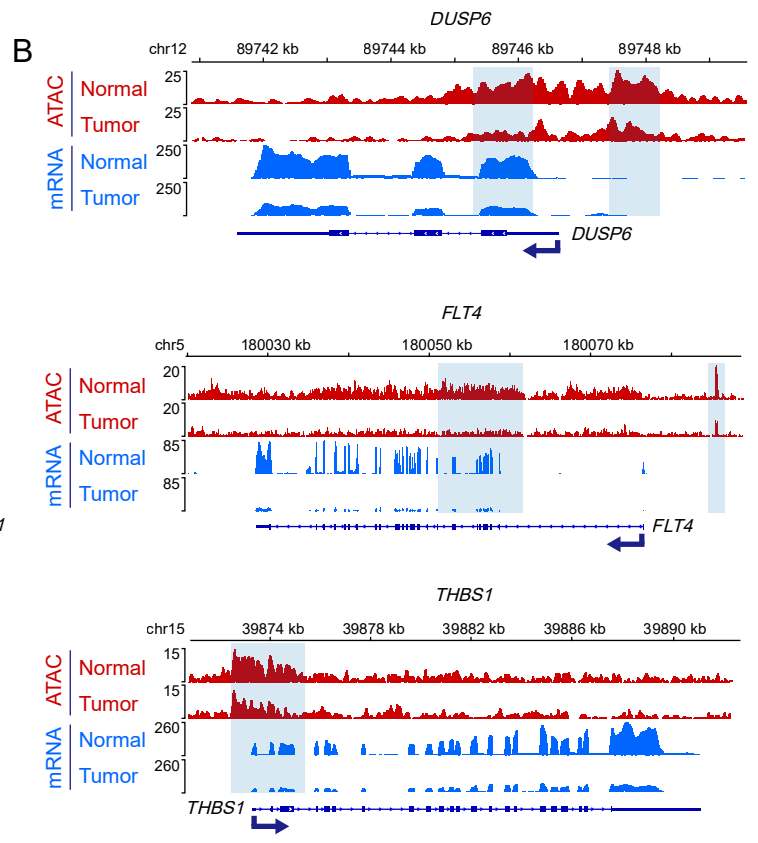

Supplement: Supplementary file 2 — Figure S1 [file 12020_2022_3155_MOESM2_ESM.pdf]
