# Supplementary material for: Global changes in chromatin accessibility and transcription in growth hormone-secreting pituitary adenoma
Source: Endocrine. 2022 Aug 10;78(2):329–42. doi: 10.1007/s12020-022-03155-z (PMC9584994; doi:10.1007/s12020-022-03155-z)
Supplement: Supplementary file 3 — Figure S2 [file 12020_2022_3155_MOESM3_ESM.docx]

**Supplemental Figures Legends**

**Fig. S1. mRNA gene expression analysis in the normal pituitary tissues and pituitary tumors.** (A) Expression levels (FPKM values) across all gene protein coding transcripts (hg19 reference genome) were used to calculate Jensen-Shannon Divergence (JSD). (B) Gene expression profiles of normal pituitary tissues (red) and pituitary tumors (green). (C) Bar plot showing the number and percentage of significantly up- (red) and down-regulated (blue) genes in pituitary tumors compared with normal pituitary tissues. (D) Heatmap showing all of 1528 DEGs.

**Fig. S2. DEGs associated with key signal pathway and positively correlated to DARs.** Genomic snapshots of ATAC-seq (red) and mRNA-seq (blue) signal of key up-regulated (A) and down-regulated (B) genes associated with key signal pathway and related to hyper- (red shade area) and hypo-accessible (blue shade area) regions, respectively.
